# Role of Pannexin 1, P2X7, and CFTR in ATP Release and Autocrine Signaling by Principal Cells of the Epididymis

**DOI:** 10.1093/function/zqaf016

**Published:** 2025-03-24

**Authors:** Kéliane Brochu, Aram Minas, Larissa Berloffa Belardin, Christine Légaré, Sylvie Breton

**Affiliations:** Faculty of Medicine, Department of Obstetrics, Gynecology and Reproduction, Centre Hospitalier Universitaire de Québec—Research Centre, and Centre de Recherche en Reproduction, Développement et Santé Intergénérationnelle—Université Laval, Québec, QC, Canada, G1V 4G2; Faculty of Medicine, Department of Obstetrics, Gynecology and Reproduction, Centre Hospitalier Universitaire de Québec—Research Centre, and Centre de Recherche en Reproduction, Développement et Santé Intergénérationnelle—Université Laval, Québec, QC, Canada, G1V 4G2; Department of Surgery, Division of Urology, Human Reproduction Section, São Paulo Federal University, São Paulo, Brazil, 04024-002; Faculty of Medicine, Department of Obstetrics, Gynecology and Reproduction, Centre Hospitalier Universitaire de Québec—Research Centre, and Centre de Recherche en Reproduction, Développement et Santé Intergénérationnelle—Université Laval, Québec, QC, Canada, G1V 4G2; Faculty of Medicine, Department of Obstetrics, Gynecology and Reproduction, Centre Hospitalier Universitaire de Québec—Research Centre, and Centre de Recherche en Reproduction, Développement et Santé Intergénérationnelle—Université Laval, Québec, QC, Canada, G1V 4G2; Faculty of Medicine, Department of Obstetrics, Gynecology and Reproduction, Centre Hospitalier Universitaire de Québec—Research Centre, and Centre de Recherche en Reproduction, Développement et Santé Intergénérationnelle—Université Laval, Québec, QC, Canada, G1V 4G2

**Keywords:** ATP release, epididymal principal cells, pannexin-1, P2X7 receptors, purinergic signaling, male reproductive tract, male reproductive health, luminal acidification, sodium reabsorption

## Abstract

Extracellular adenosine triphosphate (ATP) is a signaling molecule that acts as a paracrine and autocrine modulator of cell function. Here, we characterized the role of luminal ATP in the regulation of epithelial principal cells (PCs) in the epididymis, an understudied organ that plays crucial roles in male reproduction. We previously showed that ATP secretion by PCs is part of a complex communication system that ensures the establishment of an optimal luminal acidic environment in the epididymis. However, the molecular mechanisms regulating ATP release and the role of ATP-mediated signaling in PCs acidifying functions are not fully understood. In other cell types, pannexin 1 (PANX-1) has been associated with ATP-induced ATP release through the interaction with the purinergic P2X7 receptor. Here, we show that PANX-1 and P2X7 are located in the apical membrane of PCs in the mouse epididymis. Functional analysis using the immortalized epididymal PC cell line (DC2) and the mouse epididymis perfused *in vivo* showed that (1) PANX-1 and P2X7 participate in ATP release by DC2 cells, together with cystic fibrosis transmembrane conductance regulator (CFTR); (2) several ATP-activated P2Y and P2X purinergic receptors are expressed in DC2 cells; (3) the nonhydrolyzable ATP analog ATPγS induces a dose-dependent increase in intracellular Ca^2+^ concentration in DC2 cells, a process that is mainly mediated by P2X7; and (4) perfusion of the epididymal lumen *in vivo* with ATPγS induces the internalization of apical sodium-hydrogen exchanger 3 (NHE3) in PCs. Altogether, this study shows that luminal ATP, regulated by CFTR, PANX-1, and P2X7, modulates sodium-proton exchange in PCs in an autocrine manner through activation of purinergic receptor–mediated intracellular calcium signaling.

## Graphical Abstract

**Graphical Abstract:**
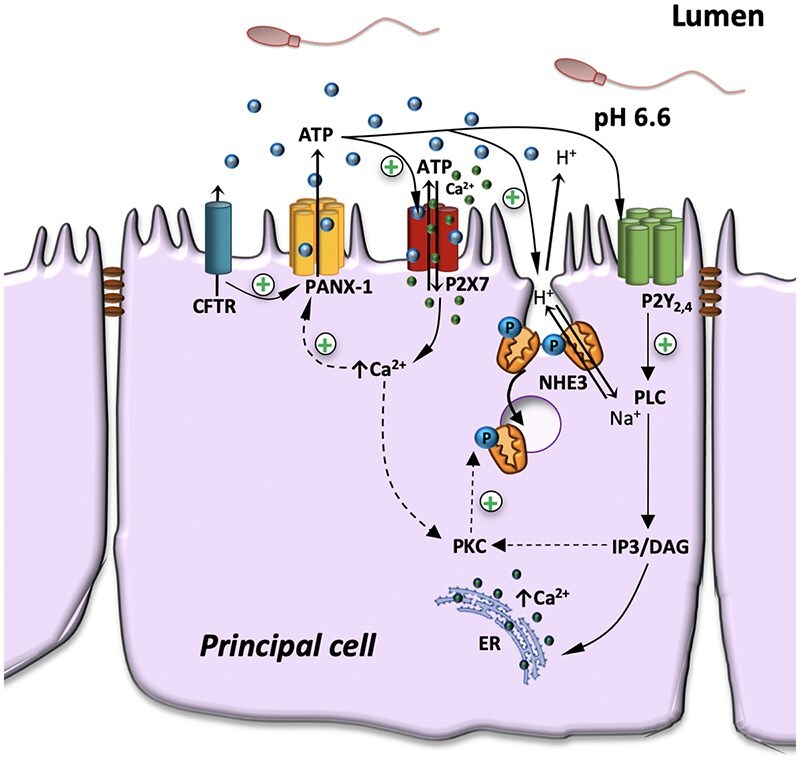
Epididymal principal cells (PCs) constitutively secrete adenosine triphosphate (ATP) into the lumen through the concerted interaction between CFTR, PANX-1, and P2X7. Activation of P2X7 by luminal ATP further activates PANX-1, providing a positive feedback mechanism for ATP release. P2X7 is an ATP-gated receptor that induces the entry of Ca^2+^ from the luminal space into the cell. P2Y receptors were also identified in PCs. Upon activation with luminal ATP, these GPCR receptors induce the release of calcium from intracellular stores. *In vivo* luminal perfusion of the cauda epididymis with ATPγS, the nonhydrolyzable form of ATP, induced the internalization of NHE3 from the apical membrane of PCs, showing the autocrine regulation of PCs. The full arrows indicate the results obtained in the present study. The dashed arrows indicate proposed mechanisms of action.

## Introduction

Adenosine triphosphate (ATP) signaling is involved in the paracrine and autocrine regulation of epithelial function.^1–14^ Extracellular ATP activates purinergic receptors, including P2X ionotropic and P2Y metabotropic receptors. These receptors are located either on the apical or basolateral membrane of epithelial cells, which complicates the study of their respective roles in physiological and pathophysiological conditions in the intact tissue. Here, we characterized how luminal purinergic signaling modulates the function of epithelial cells lining the epididymis, a small organ located downstream of the testis that plays crucial roles in the establishment of male fertility.

The epididymis is anatomically divided into distinct segments, each with specific functional roles. In rodents, it consists of 4 segments, including the initial segment, caput, corpus, and cauda,^15–19^ while in humans, the initial segment is absent and the epididymis is composed of the caput, corpus, and cauda.^20,21^ Each segment is lined by a pseudostratified epithelium containing principal cells (PCs), clear cells (CCs), and basal cells (BCs).^15,17,18,20,22^ These cells work in a concerted manner to establish a unique environment that is optimal for the concentration, protection, maturation, and storage of spermatozoa in the post-testicular tract.^15,16,23–25^

The luminal environment of the epididymis is characterized by high osmolarity, elevated potassium, low sodium and low bicarbonate concentrations, and an acidic pH.^25–27^ Impairment of luminal acidification in the epididymis can lead to male infertility by preventing spermatozoa from moving up the female reproductive tract and interacting with the oocyte.^28,29^ To maintain this acidic environment, the epididymal epithelium establishes complex communication networks that coordinate interactions between epithelial cells, allowing them to respond to and modulate their immediate environment.^16^ Specifically, epididymal luminal acidification depends on the interactions between PCs, CCs, and BCs, through extracellular mediators that are secreted by either PCs or BCs to modulate proton secretion by CCs.^2,22,30,31^ One such mediator is ATP, which is secreted by PCs^30^ and induces the apical accumulation of the proton pump V-ATPase in the apical membrane of CCs.^22,32^ Luminal acidification maintains spermatozoa in a dormant state during their storage in the epididymis,^33–35^ and facilitates the transfer of protein and small RNAs to spermatozoa via extracellular vesicles, called epididymosomes, that are secreted by epithelial cells.^36–39^ We recently showed that, in addition to CCs, PCs contribute to the establishment of an acidic luminal environment in the epididymis through their expression of the sodium-proton exchanger type 3 (NHE3 or SLC9A3) in their apical membrane.^40,41^ NHE3 facilitates sodium reabsorption and luminal acidification in several tubular organs,^42^ by exchanging one luminal sodium ion for one intracellular proton. In the epididymis, NHE3 is located in long actin-rich apical extensions of PCs, called stereocilia,^43,44^ whereas in other organs, such as the kidney and the intestine, NHE3 is localized in actin-rich brush-border membrane.^42^ In these organs, NHE3 is also located intracellularly and its membrane expression is regulated through vesicle recycling, intracellular protein transport, interactions with regulatory proteins, and direct protein phosphorylation. Activation of Ca^2+^, cAMP, and cGMP signaling pathways inhibits NHE3-dependent sodium/proton exchange by inducing its internalization through clathrin-mediated endocytosis. Activation of P2X and P2Y receptors by extracellular ATP induces an increase in intracellular Ca^2+^ concentration.^2,13,45,46^ Here, we explore the direct role of ATP in NHE3 regulation via an autocrine mechanism following ATP secretion by PCs.

ATP release occurs through various transport mechanisms, including exocytosis and permeation through conductive pores.^47,48^ Initially, the cystic fibrosis transmembrane conductance regulator (CFTR) was suggested to function as an ATP channel, but it was later demonstrated that it modulates a separate ATP release pathway (reviewed in Praetorius and Leipziger^45^). In the epididymis, CFTR is highly expressed in the epithelium,^49–52^ where it functions as an anion channel that participates in chloride and bicarbonate secretion.^53,54^ More recently, we showed that CFTR can also act as a modulator of separate transport pathways in epididymal PCs, such as water transport through aquaporin 9^51^ or ATP release.^30^ However, the exact mechanisms of ATP release by epididymal PCs and their regulation through purinergic signaling remain unclear. Pannexin 1 (PANX-1) channels and P2X7 receptors (P2X7R) were shown to be involved in ATP release across various tissues^1,55^ (reviewed in Di Virgilio et al.^56^ and Dahl^57^).

In this study, using a multidisciplinary approach that combines immortalized epididymal PC cell line (DC2), and perfused epididymis *in vivo*, we show that PCs secrete ATP through the combined action of CFTR, PANX-1, and P2X7. We found that ATP participates in the autocrine regulation of PCs through intracellular calcium signaling, which induces NHE3 internalization. Our study, thus, provides evidence for the autocrine regulation of PCs acidifying function through luminal ATP–mediated purinergic signaling. Such local regulation by ATP could be extrapolated to other tubular organs, such as the kidneys, the lungs, and the intestine, which similarly rely on ATP signaling to control their function.

## Materials and Methods

### Ethics Approval and Animals

Adult male C57Bl/6Ncrl mice, aged 10–12 weeks, were obtained from Charles River Laboratories (Sherbrooke, QC, Canada). Previously generated V-ATPase B1-EGFP (B1-EGFP) mice were used.^58^ All animal procedures in this study were reviewed and approved by the research ethics committee of the Institutional Review Board at the Centre Hospitalier Universitaire de Québec (CHU de Québec), and were conducted in accordance with the Guide for the Care and Use of Laboratory Animals. The mice were bred and housed in the Animal Facility at the CHU de Québec Research Center—Université Laval.

### Chemicals and Reagents

The CFTR inhibitor CFTR inhibitor 172 (CFTR_inh172_; cat # 219670), the pannexin-1 channel inhibitor probenecid (PBN; cat # P8761), and the P2X4 antagonist PSB-12062 10-[(4-methylphenyl)sulfonyl]-10H-phenoxazine (cat # SML0753) were obtained from Sigma (St Louis, MO, USA). The general P2 purinergic antagonist PPADS [pyridoxal-phosphate-6-azophenyl-20,40-disulfonic acid tetrasodium salt] (cat # 0625), the P2X7 agonist BzATP [2″(3″)-O-(4-benzoylbenzoyl)adenosine-5′-triphosphate tri(triethylammonium) salt] (cat # 3312), the P2X7 antagonist A438079 3-[[5-(2,3-dichlorophenyl)-1H-tetrazol-1-yl]methyl]pyridine hydrochloride] (cat # 2972), and ATPγS [adenosine 5′-(γ-thio)triphosphate tetralithium salt] (cat # 4080) were obtained from Tocris Bioscience (Burlington, ON, Canada).

### Antibodies Used for Western Blotting and Immunofluorescence

Table 1 provides a list of the primary antibodies and secondary antibodies, including their respective concentrations and the immunizing peptides used as negative controls. For immunofluorescence, primary and secondary antibodies were diluted in Dako antibody diluent (S0809, Dako, Glostrup, Hovedstaden, Denmark).

**Table 1.** List of Antibodies

Antibody	Host Species	Conjugate	Company	Catalog Number	Application	Concentration
	**Primary antibodies**					
PANX-1	Rabbit	Unconjugated	Alomone Labs (Jerusalem, Israel)	ACC-234	WB; IF	0.8 µg/mL; 4 µg/mL
P2X7	Rabbit	Unconjugated	Alomone Labs	APR-004	WB; IF	0.2 µg/mL; 3 µg/ml
	**Secondary antibodies**					
Anti-rabbit	Goat	Horseradish peroxidase (HRP)	Jackson ImmunoResearch (Westgrove, PA, USA)	111-035-045	WB	1:3000 (v:v)
Anti-rabbit	Donkey	AlexaFluor^TM^ 555	Invitrogen	A-31572	IF	1:500 (v:v)
	**Peptides**					
PANX-1	N/A	Unconjugated	Alomone Labs	BLP-CC234	WB; IF	4 µg/mL; 20 µg/mL
P2X7	N/A	Unconjugated	Alomone Labs	BLP-PR004	WB; IF	2 µg/mL; 30 µg/mL

### Cell Culture

The immortalized principal epididymal cell line, DC2 (Distal Caput-2), generously provided by Dr Marie-Claire Orgebin-Crist, was cultured as previously described.^59^ The DC2 cells were maintained in Iscove’s Modified Dulbecco’s Medium (IMDM, Multicell Wisent Inc., Saint-Jean-Baptiste, Canada) supplemented with 5α-dihydrotestosterone (1 n m) and 10% fetal bovine serum (Multicell Wisent Inc.) at 33°C. These cells are representative of epididymal PCs. For immunofluorescence labeling, the cells were fixed with 4% paraformaldehyde (PFA) in phosphate buffered saline (PBS) for 20 min and permeabilized with 1% sodium dodecyl sulfate (SDS) and 0.1% Triton X-100 in PBS for 4 min. Primary and secondary antibodies were applied as described later. For reverse transcription-polymerase chain reaction (RT-PCR) analysis, DC2 cells were resuspended in PBS and centrifuged at 400 *g* for 5 min. The pelleted DC2 cells were washed 2 times with PBS, and the pellet was then stored at −80°C until used.

### *In Vivo* Perfusion of the Mouse Cauda Epididymis

Due to its relatively large diameter compared to other tubular organs such as the kidney, the cauda epididymis can be perfused luminally *in vivo*, thus allowing the study of epithelial cells by luminal factors, independently from basolateral stimuli. Here, in vivo luminal perfusion of the distal cauda epididymis was performed in mice anesthetized with ketamine/xylazine (100 and 10 mg/kg, respectively), as described previously.^22,30,31,40,41^ We focused on this region due to its larger lumen diameter for *in vivo* perfusion, unlike the narrower epididymal proximal regions. Briefly, the cauda epididymis was exposed by an abdominal incision, under a dissecting microscope, and the vas deferens was cannulated with a microcannula (0.61 mm O.D., 0.28 mm I.D., Warner Instruments, Hamden, CT, USA). The distal cauda was perfused by inserting a homemade catheter into the lumen of the tubule. Perfusion was conducted (0.25 mL/h) for 20 min with a physiological solution (50 m m NaCl, 50 m m K-gluconate, 1.2 m m MgSO_4_, 0.6 m m CaCl_2_, 4 m m Na acetate, 1 m m Na_3_ citrate, 6.4 m m NaH_2_PO_4_, and 3.6 m m Na_2_HPO_4_, adjusted to 350–360 mosmol/kg with raffinose) adjusted at the alkaline pH of 7.8, with or without 100 µm ATPγS.

After the experimental phase, the cauda lumen was fixed with 4% PFA in PBS for 20 min. Caudae were further fixed by immersion in 4% PFA overnight at 4°C. Tissues were cryoprotected in PBS containing 30% sucrose and 0.02% sodium azide for 48 h at 4°C. Subsequent embedding in Tissue-Tek OCT compound (Sakura Finetek, Torrance, CA, USA), mounting, and freezing on a cryostat cutting block (Leica CM3050-S) were performed. Sections were cut at 10 μm thickness, picked up onto Fisher Superfrost/Plus microscope slides (Fisher Scientific), and stored refrigerated until used.

### Western Blotting

Total protein extracts (30 μg) from epididymis tissues and DC2 cell samples were separated by electrophoresis under denaturing conditions using 8%–12% polyacrylamide gels.^60^ After sodium dodecyl sulfate polyacrylamide gel electrophoresis (SDS-PAGE) separation, proteins were transferred onto nitrocellulose membranes using a semi-dry transfer system (Trans-Blot Turbo, Bio-Rad). Membranes were immunoblotted with the primary antibody overnight at 4°C. Membranes were washed 3 times for 15 min each time with PBS and 0.1% Tween-20 (PBS-T) and then incubated with the secondary antibody for 1 h at room temperature. After 3 additional washes, bands were detected using the chemiluminescent Clarity Western ECL substrate (170-5061; BioRad). Images were acquired with a ChemiDoc MP Image system (Bio-Rad, Berkeley, CA, USA).

### Reverse Transcription-Polymerase Chain Reaction

RT-PCR analysis were performed as previously described.^61^ Briefly, frozen tissues from mouse caput epididymis were ground to a powder in liquid nitrogen. RNA was extracted from epididymal tissues and pelleted DC2 cells with TRIzol reagent (Invitrogen, Carlsbad, CA), following the manufacturer’s instructions. RNA was dissolved in DEPC-treated sterile water and quantified using a NanoDrop ND-1000 spectrophotometer (NanoDrop Technologies, Wilmington, DE, USA). RNA integrity was assessed by agarose gel electrophoresis. The RNA was then reverse-transcribed using Superscript II enzyme (Invitrogen). The primers used for amplification are indicated in Table 2. The PCR products were resolved on a 1.5% or 2% agarose gel, depending on the fragment size, and sent for sequencing at the CHUQ Research Center Core Facility (Quebec, QC, Canada).

**Table 2.** List of Primers Used for Reverse Transcription-Polymerase Chain Reaction

Target Genes	Primers		Annealing Temperature (°C)	Size (bp)
	Forward	Reverse		
*P2X1*	GAC AAA CCG TCG TCA CCT CT	CCC ATG TCC TCC GCA TAC TT	55	222
*P2X2*	GTT CTG GGA CTA CGA GAC GC	GCT GTG AAC CCT CAT GCT CT	52	328
*P2X3*	ACC AAG TCG GTG GTT GTG AAA	TGT TGG CAT AGC GTC CGA AG	55	181
*P2X4*	GGC CAC AGC TTT CAG GAG AT	CAA ACT TGC CAG CCT TTC CA	55	274
*P2X5*	AGT TAA TGG CAA GGC GGG AA	TCT CCT GGA GGC CAG ACC	55	347
*P2X6*	TCA GGC CAA GAA CTT CAC AC	CCA GGT TGC AAT CCC AGT GA	52	251
*P2X7*	GCA CGA ATT ATG GCA CCG TC	CCC CAC CCT CTG TGA CAT TC	55	170
*P2Y1*	AGT ACT GTC GCC TCA ACT GC	GCA GCT TGC ACA TAG CAT CC	55	298
*P2Y2*	ATC ACT TGA CCT CAG CTG CC	GTC GTC ACT GCT GAC TGA CA	55	250
*P2Y4*	CTG GCA AGA CTG TTG AAC GC	AGG GAG GAA GCA GTT GTT CG	55	177
*P2Y6*	TGC CAA TCT ACA TGG CAG CA	AGC GAG TAG ACA GGA TGG GT	55	225
*P2Y12*	TTGCACGGATTCCCTACACC	ATT GGG GTC TCT TCG CTT GG	55	262
*P2Y13*	TCAGTCACACCTGCCAGTTC	TGG GGC AAA GCA GAC AAA GA	55	182
*P2Y14*	CCATGCAAAATGGAAGTCTG	CGG AAA GAC TGG GTG TCT TC	52	147

### Immunofluorescence Labeling

Immunofluorescence was performed according to established protocols.^22,40,41,62^ Briefly, cryosections of the luminally perfused distal cauda epididymis, or DC2 cells were rehydrated in PBS for 15 min, followed by a permeabilization/antigen retrieval step with SDS 1%-Triton X-100 0.1% in PBS for 4 min. The slides were then blocked with 1% BSA for 30 min at room temperature and incubated overnight at 4°C with primary antibodies. Afterward, the slides were washed twice with high-salt PBS (2.7% NaCl w/v) and once with PBS, each for 5 min. Secondary antibodies were then applied and incubated for 1 h at room temperature, followed by the same washing steps as for the primary antibodies. For negative controls, the primary antibody was preadsorbed with its corresponding immunizing peptide, as specified in Table 1, following the manufacturer’s recommendations.

Finally, the slides were mounted with Vectashield mounting medium containing DAPI (4',6-diamidino-2-phenylindole) (Vector Laboratories, Burlingame, CA, USA). Images were acquired using a Zeiss LSM900 confocal microscope equipped with Airyscan high-resolution imaging (Zeiss Laboratories, Oberkochen, Germany), with a 20X/0.8 objective or a 40X/1.4 oil immersion objective, as indicated in the figure legends.

### ATP Release Measurement

The ATP levels secreted by DC2 cells were measured using a luciferin-luciferase–based assay (ATP Bioluminescence Assay Kit, Sigma-Aldrich, St Louis, MO, USA), as previously described.^30^ Briefly, DC2 cells were cultured to confluency in 24-well plates. Roswell Park Memorial Institute (RPMI) media, without phenol red (RPMI; Multicell Wisent Inc.), containing either the vehicle or inhibitors was gently added to each well (500 µL/well). Following a 10-min incubation at 33°C, the cell-free media was collected and heated at 99°C for 2 min to inactivate ectonucleotidases. ATP concentrations were then measured using a luciferin-luciferase–based bioluminescence assay kit, according to the manufacturer’s instructions. The luminescence was recorded using a luminometer (SparkTM 10 M, Tecan, Männedorf, Switzerland). All drugs used were tested and confirmed to have no significant effect on the bioluminescence signal. Cells in each well were lysed, and protein concentration was determined using the Bicinchoninic Acid (BCA) assay (23225; Pierce^TM^ BCA Protein Assay Kit, Thermo Fisher Scientific, Waltham, MA, USA). Each experimental group was repeated between 6 and 16 times, depending on the condition studied. For each experiment, experimental group data were normalized by their corresponding control group (control conditions without addition of inhibitors).

### Intracellular Calcium Measurement

Calcium imaging of DC2 cells was conducted using the microfluidic BioFlux 1000z imaging system (Fluxion Biosciences Inc., Oakland, CA, USA). DC2 cells were seeded into fibronectin-coated channels (FC010; 2% fibronectin (v/v), Sigma-Aldrich) of a 48-well microfluidic plate (910-0047; Fluxion Biosciences Inc.) and cultured for 24 h in IMDM media at 33°C. The following day, cells were loaded with 5 µm of the fluorescent calcium-sensitive dye Cal-520 AM (ab171868; Abcam, Toronto, ON, Canada) diluted in IMDM for 1 h at 33°C. After washing once with IMDM media, the cells rested for 15 min to allow for cleavage of the lipophilic groups of Cal-520 AM by the cell esterases, resulting in the production of the impermeant form of Cal-520. When indicated, cells were preincubated with purinergic receptor agonists and antagonists for 30 min. Real-time calcium fluctuations were monitored for 20 min, with images taken at 5-s intervals. Fluorescence images were acquired with a 20X/0.8 air objective at an excitation wavelength of 490 nm and detected at 517 nm with the automated BioFlux 1000z microscopy system mounted onto a Zeiss Observer.Z1 inverted microscope. After an initial control period (200 s), ATPγS was added into the microfluidic channel at a constant perfusion rate and the Cal-520 fluorescence was recorded for an additional period of 800 s. The effect of ATPγS was also assessed in the presence (DMEM media, Thermo Fischer) or absence of extracellular Ca^2+^ (Ca2+-free DMEM, Thermo Fischer). For the Ca^2+^-free condition, 1 m m of ethylene glycol-bis-(β-aminoethylether)-*N,N,N′,N′*-tetraacetic acid (EGTA), a calcium chelator, was added to the medium. Cells were preincubated for 20 min in the Ca^2+^-free condition prior to addition of ATPγS. To evaluate changes in Ca^2+^ fluorescence, for each experiment, the mean fluorescence intensity (MFI) was quantified using Fiji software (NIH, Bethesda, MD, USA) in 4 groups of cells (each comprising at least 15 cells), identified as regions of interest. Background fluorescence was measured in 3 regions without cells and subtracted from all measurements. Changes in the fluorescence were then normalized against the baseline, determined as the average MFI before the addition of agonists or antagonists. For each treatment, the experiment was repeated at least 4 times.

### Quantification of NHE3 Accumulation in PC Apical Stereocilia

The effect of ATPγS on NHE3 subcellular localization was quantified from the cauda epididymis luminally perfused *in vivo*. First, to visualize NHE3 localized either in the apical stereocilia or the intracellular compartment in PCs, 3D reconstruction projections from confocal images of NHE3 labeling were acquired along the Z axis at 0.45 µm interval. The area occupied by NHE3 in the stereocilia of PCs was then quantified from immunofluorescence single-Z confocal images focused on the stereocilia area, as we recently published.^40^ Images were merged with bright-field images to visualize stereocilia, and the total area of the stereocilia was quantified using Fiji software (NIH, Bethesda, MD, USA). The area occupied by NHE3-associated fluorescence within the stereocilia was determined using the “Color Threshold” tool. The area occupied by NHE3 in the stereocilia was then divided by the total area of the stereocilia. Data are expressed as the percentage of the stereocilia area occupied by NHE3-associated fluorescence signal. Each experimental group included 5 epididymides. At least 100 cells were quantified per group (with approximately 20 cells analyzed per epididymis).

### Statistical Analysis

Numeric data were analyzed using GraphPad Prism 9 software (version 9.3.1) (La Jolla, CA, USA). Outliers were identified using ROUT with Q set at 0.05%. In order to compare the effect of treatments between two groups, the Mann-Whitney nonparametric test was performed. Comparison of treatments (fold change) with their respective control was determined using a 1-sample *t*-test (where control = 1). For multiple groups, data were analyzed using Welch (analysis of variance) ANOVA with Dunnett’s T3 multiple comparisons test or using 1-way ANOVA followed by a Tukey’s post hoc test. Data are shown as box plots (min to max whiskers) and statistical significance was set at *P* < .05.

## Results

### Apical Localization of PANX-1 and P2X7 in PCs of the Mouse Epididymis

**Figure 1.**
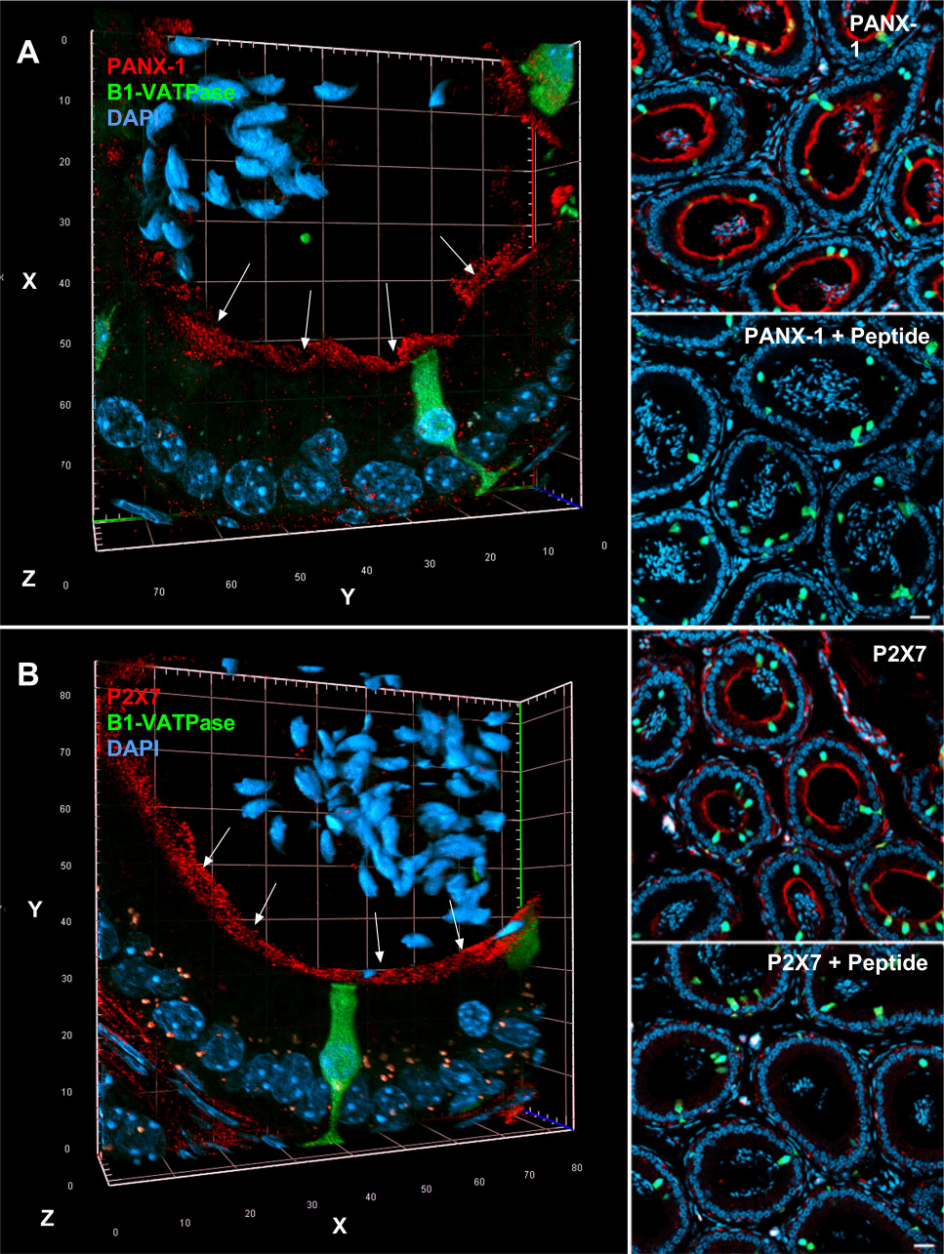
Confocal microscopy showing apical localization of pannexin 1 (PANX-1) channel and P2X7 receptor in principal cells (PCs) of the mouse epididymis. 3D reconstruction of confocal microscopy images for PANX-1 channel (A; left panel) and ATP-gated P2X7 receptor (B; left panel) labelled in red in the mouse epididymis. V-ATPase-positive clear cells (CCs), which specifically express EGFP under the promoter of the B1 subunit (B1-VATPase), can be visualized in green. Both PANX-1 and P2X7 are enriched in the apical membrane of PC (arrows, EGFP-negative cells). PANX-1 and P2X7 labeling was abolished when the antibody was preincubated with the immunizing peptide (right panels). Nuclei are labelled with DAPI in blue. Images were acquired with a 20X/0.8 objective. (Left panels) Each box = 20 µm^2^. (Right panels) Scale bars = 20 μm.

There are several transporters and channels that have been shown to be involved in ATP release. Here, we focus on the potential role of PANX-1 channels and ATP-gated P2X7 receptor in this process. We first examined their expression in the mouse epididymis by immunofluorescence labeling. 3D reconstruction confocal microscopy imaging showed a strong expression of PANX-1 (Figure 1A), corroborating previous studies,^30,63^ as well as P2X7 (Figure 1B) in the apical membrane of epididymal PCs (red). No labelling was detected in CCs (identified by their positive V-ATPase-driven EGFP labeling (green). Cells located in the interstitium also showed the expression of P2X7. These cells were not identified in this study. Several spermatozoa, labeled in blue with DAPI, are shown in the epididymal lumen. The PANX-1 and P2X7 staining was abolished when the antibodies were preadsorbed with their respective immunizing peptides, confirming the specificity of the immunolabeling (Figure 1A and B; right panels).

### Epididymal PC Line DC2 Expresses PANX-1 Channel and P2X7 Receptor

**Figure 2.**
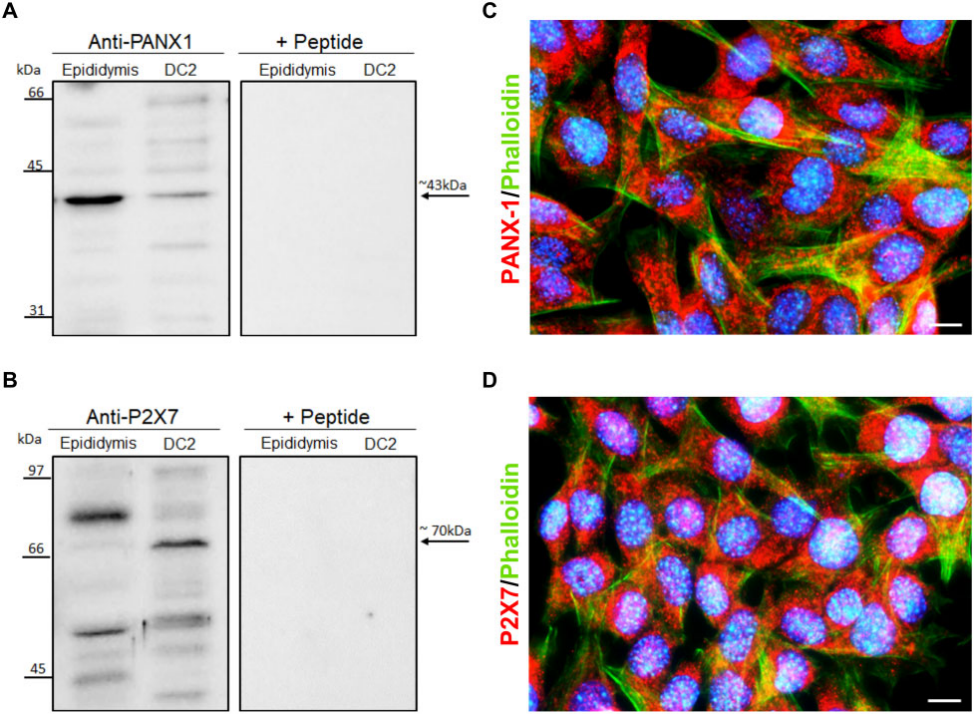
Expression of pannexin 1 (PANX-1) channel and P2X7 receptor by DC2 cells. Western blot detection of PANX-1 (A: left panel) and P2X7 (B: left panel) in DC2 cells and in the mouse epididymis (used as a positive control). Preadsorption of antibodies with their respective immunizing peptide abolished the signal detected in DC2 cells and in epididymis extracts (right panels). Immunofluorescence labeling microscopy images showing the expression of PANX-1 (C) and P2X7 (D) in red in DC2 cells. Actin filaments of DC2 cells are stained in green with phalloidin. Nuclei are labeled with DAPI in blue. Images were acquired with a 40X/1.4 oil immersion objective. Scale bars = 20 μm.

To investigate the involvement of PANX-1 channel and P2X7 receptor in ATP release from epithelial cells, we used the previously established DC2 mouse epididymal PC line.^59^ Western blot analysis revealed a ∼43-kDa band (Figure 2A, left panel) and a ∼70-kDa band (Figure 2B, left panel) in DC2 cells, corresponding to the molecular sizes of PANX-1 and P2X7, respectively. Similar bands were also observed in mouse epididymis tissues, which served as a positive control.

For the anti-P2X7 antibody, additional bands corresponding to several P2X7 splice variants were observed, consistent with previous findings in other organs.^56,64–67^ The specificity of the anti-PANX-1 and anti-P2X7 antibodies was confirmed by the absence of signal when the antibodies were preincubated with their respective immunizing peptides (Figure 2A and B; right panels).

Additionally, positive immunofluorescence labeling was obtained using the same anti-PANX-1 (Figure 2C; red) and anti-P2X7 (Figure 2D; red) antibodies in DC2 cells. The actin cytoskeleton was labeled with phalloidin in green, and nuclei are visualized in blue with DAPI. Altogether, these results show that DC2 cells express PANX-1 channel and P2X7 receptor.

### Participation of CFTR, PANX-1, and P2X7 in ATP Release From DC2 Cells

**Figure 3.**
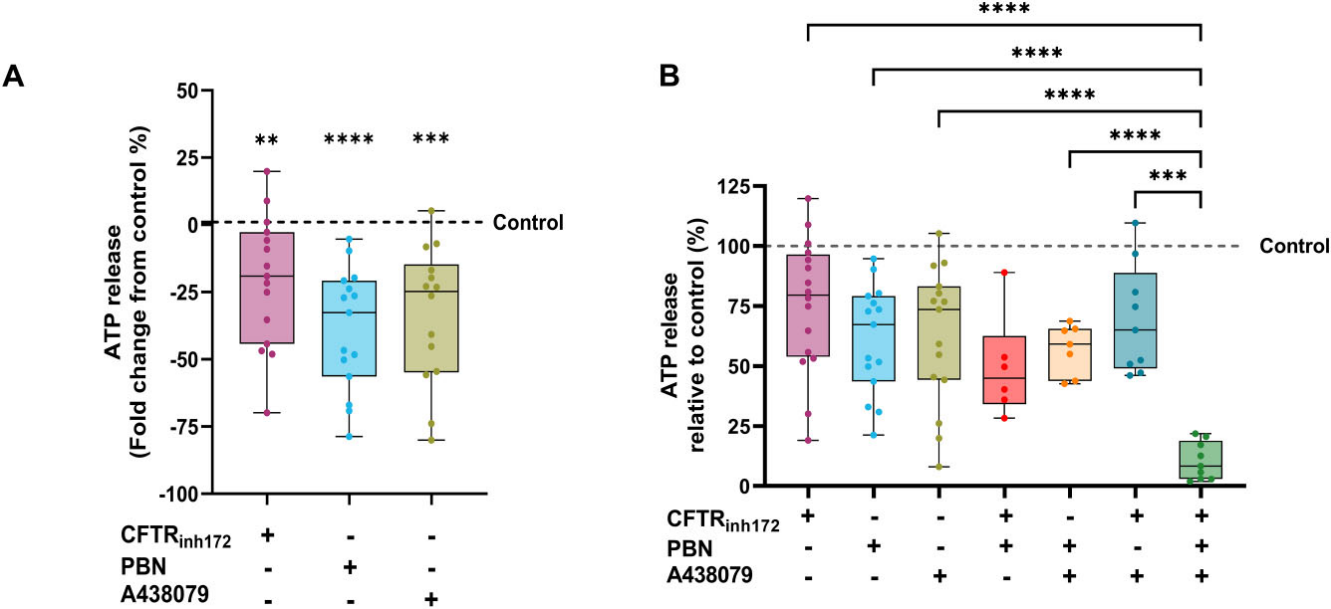
Role of cystic fibrosis transmembrane conductance regulator (CFTR), pannexin 1 (PANX-1), and P2X7 in ATP release from DC2 cells. Effect of the CFTR inhibitor CFTR_inh172_ (10 μm), the PANX-1 inhibitor probenecid (PBN; 300 µm), and the P2X7 antagonist A438079 (75 µm) on ATP release from DC2 cells. Baseline ATP secretion was detected under control conditions. (A) Treatments with CFTR_inh172_, PBN, or A438079 significantly reduced baseline ATP secretion. (B) The effects of combined treatments were then compared to the effect elicited by each inhibitor when applied individually. Here, the 3 first experimental groups are the same as shown in panel A, to allow this comparative analysis. No additional effect was detected when inhibitors were applied in pairs (CFTR_inh172_ + PBN, PBN + A438079, or CFTR_inh172_ + A438079). In contrast, when all 3 inhibitors were applied together (CFTR_inh172_ + PBN + A438079), basal ATP release levels were almost completely inhibited. Experimental groups were normalized by the mean of their corresponding control group. Data are expressed as fold change from control (A) or as ATP release relative to control (B). Data are shown as box and whiskers (min to max); *n* = 6–16. Each dot represents one experiment. ^∗∗^*P* < .01; ^∗∗∗^*P* < .001; ^∗∗∗∗^*P* < .0001 by a 1-sample *t*-test (A), or Welch ANOVA with Dunnett’s post hoc test (B).

After confirming the protein expression of PANX-1 and P2X7 in DC2 cells, we investigated their role in ATP release using specific modulators. Using a luciferin-luciferase assay, we showed basal ATP secretion in the DC2 culture medium following a 10-min incubation under control conditions. Cells were then treated with previously characterized inhibitors of CFTR (CFTR_inh172_; 10 μm), PANX-1 (probenecid, PBN; 300 μm), and P2X7 receptor (A438079; 75 μm).^68,69^ When applied individually, each inhibitor significantly decreased ATP levels, compared to control (Figure 3A). In addition, to assess whether CFTR is involved in the regulation of ATP release mediated by PANX-1 and P2X7, we applied their specific inhibitors simultaneously (Figure 3B). When applied in pairs (Figure 3B; CFTR_inh172_ + PBN, PBN + A438079, or CFTR_inh172_ + A438079), no additional effect was observed compared to the inhibitors applied separately. However, basal ATP release was nearly completely blocked when all 3 inhibitors (Figure 3B; CFTR_inh172_ + PBN + A438079) were applied together, indicating the participation of CFTR, PANX-1 channels, and P2X7 receptors in regulating ATP secretion by DC2 cells.

### Expression of ATP-Sensitive P2 Receptors in Epididymal DC2 Cell Line

**Figure 4.**
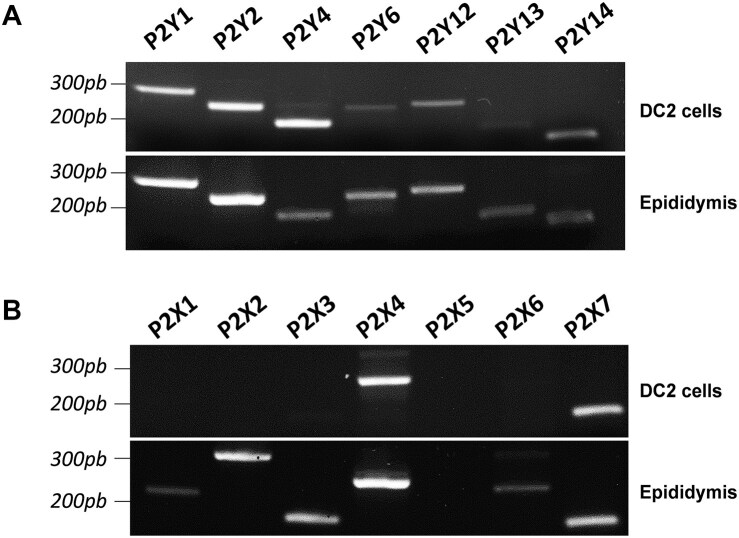
Expression of P2 receptors in DC2 cells and in the mouse epididymis. Reverse transcription-polymerase chain reaction (RT-PCR) analysis of P2Y receptors (A) and P2X receptors (B) showed mRNA expression in DC2 cells (top panels), and mouse epididymis (bottom panels). All P2Y receptors tested were expressed in both DC2 cells and epididymis. In contrast, only P2X4 and P2X7 were detected in DC2 cells, while P2X1, P2X2, P2X3, P2X4, P2X6, and P2X7, but not P2X5, were detected in the mouse epididymis.

To characterize the response of PCs to extracellular ATP, we first examined the expression of ATP-sensitive P2 receptors by RT-PCR analysis in DC2 cells and epididymis.^30,59^ All subtypes of the P2Y receptor family were detected in DC2 cells (Figure 4A; top panel) and mouse epididymis extract (Figure 4A; bottom panel). Additionally, only P2X4 and P2X7 subtypes were detected in DC2 cells (Figure 4B; top panel), while 6 P2X receptor subtypes were detected in the mouse epididymis, including P2X1, P2X2, P2X3, P2X4, P2X6, and P2X7 (Figure 4B; bottom panel). P2X5 was absent in DC2 cells and epididymis.

### Extracellular ATPγS Promotes Calcium Signaling in DC2 Cells Through Activation of Both P2X and P2Y Receptors

**Figure 5.**
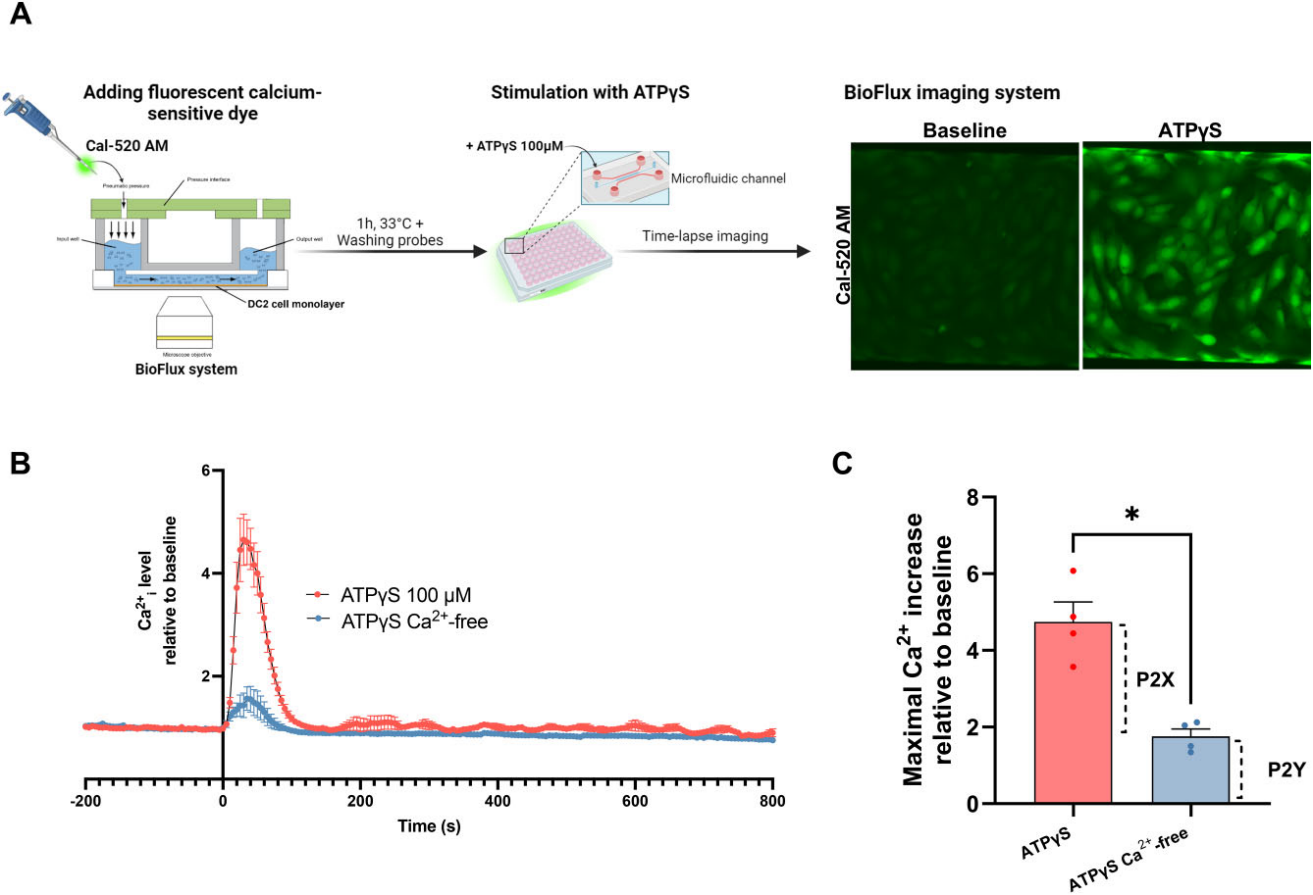
ATP-induced intracellular Ca^2+^ increase is mediated by both P2X and P2Y receptors’ activation in DC2 cells. (A) Schematic representation of the calcium fluorometric assay in DC2 cells using the microfluidic BioFlux imaging system (left panel). Real-time ATP-induced Ca^2+^_i_ response was measured over time using the fluorescent calcium-sensitive dye, Cal-520 AM (5 µm). Images in the right panels show Cal-520 fluorescence before (baseline) and after addition of ATPγS. (B) Stimulation with 100 µm ATPγS, a nonhydrolyzable form of ATP, triggered a rapid and transient Ca^2+^_i_ increase (ATPγS). This ATP-elicited calcium response was greatly reduced in the absence of external calcium (Ca^2+^-free solution + EGTA 1 m m; ATPγS Ca^2+^-free). (C) Comparison of the maximal Ca^2+^_i_ response in the presence or absence of external Ca^2+^ revealed a significant decrease under Ca^2+^-free conditions. Intracellular Ca^2+^ level was normalized for baseline (corresponding to the values before the addition of ATPγS). Data are shown as mean ± SEM (*n* = 4). ^∗^*P* = .0286 by the Mann-Whitney nonparametric test.

To explore the potential involvement of ATP-sensitive P2 receptors in ATP-mediated Ca^2+^ signaling in PCs, we performed real-time calcium imaging analysis. The microfluidic BioFlux 1000z System technology was used to examine the effect of ATPγS on the intracellular concentration of Ca^2+^ (Ca^2+^_i_) in DC2 cells (Figure 5A; refer to the “Materials and Methods” section for further details). Injection into the fluidic channel of the nonhydrolyzable ATP analog ATPγS (100 µm to achieve maximum ATP signaling activation) evoked a rapid and transient increase in Ca^2+^_i_ (Figure 5B). However, in the absence of external Ca^2+^, the ATPγS-elicited calcium response was greatly diminished (Figure 5B). The significant decrease in the Ca^2+^_i_ response under extracellular Ca^2+^-free conditions, compared to the response observed in the presence of extracellular Ca^2+^ (Figure 5C), suggests a primary role for ionotropic P2X receptors in mediating this effect, with a partial contribution from metabotropic P2Y receptors.

**Figure 6.**
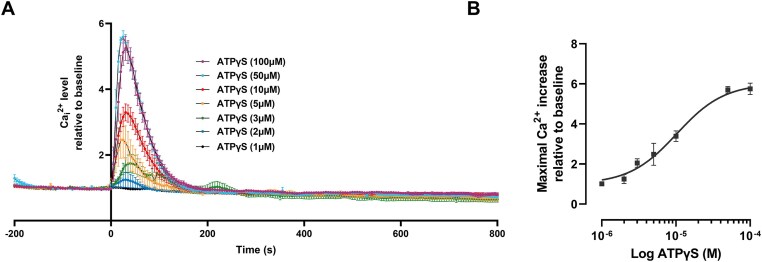
Concentration curve response of the ATPγS-evoked calcium response in DC2 cells. (A) Representative traces with mean ± SEM of ATPγS-induced Ca^2+^_i_ increase in DC2 cells, obtained with extracellular ATPγS stimulation with the concentration range of 1–100 µm. (B) Maximal Ca^2+^_i_ response was used to produce a dose-response curve of DC2 cells to ATPγS, where EC_50_ of ATPγS = 10.26 µm (fitting the Hill equation).

Figure 6 shows the dose-dependent effect of extracellular ATPγS (1–100 µm) on Ca^2+^_i_ in DC2 cells (Figure 6A and B), corresponding to an EC_50_ of 10.3 µm. Based on these results, the effect of purinergic P2 receptor agonists and antagonists was examined at the ATPγS concentration of 10 µm.

### ATP-Gated P2X7 Receptor Is Involved in ATP-Induced Calcium Signaling in DC2 Cells

**Figure 7.**
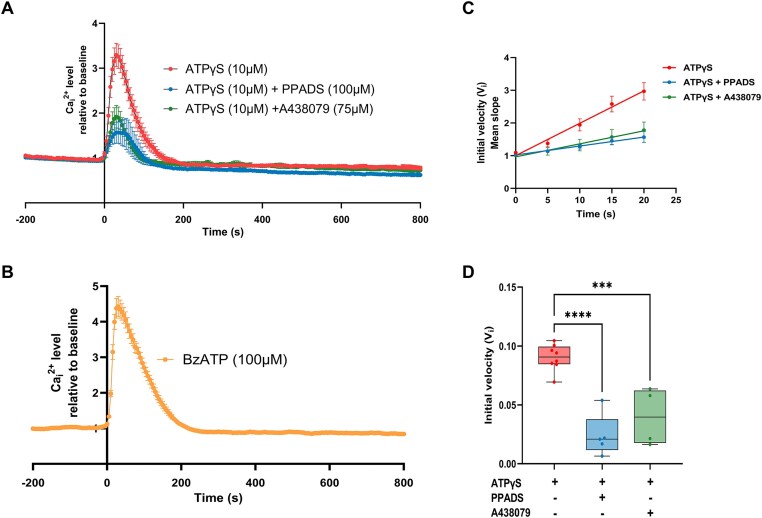
Participation of P2X7 receptors in the ATPγS-induced Ca^2+^ response in DC2 cells. (A) Representative traces of the Ca^2+^_i_ increase observed upon stimulation with extracellular ATPγS (10 µm), or with ATPγS + the general P2 receptor antagonist, PPADS (100 µm), or ATPγS + the selective P2X7 receptor antagonist, A438079 (75 µm). DC2 cells were preincubated with the antagonists for 30 min before stimulation with ATPγS. A significant reduction in the ATP-evoked Ca^2+^_i_ response was observed in the presence of PPADS or A438079 (75 µm) compared to ATPγS alone. (B) Representative traces of the Ca^2+^_i_ increase observed upon stimulation with the selective P2X7 agonist BzATP (100 µm). (C and D) A significant decrease of the initial velocity (V_i_) of the ATP-evoked Ca^2+^_i_ response was observed in presence of PPADS or A438079. Data are shown as box and whiskers (min to max); *n* = 4–8. ^∗∗∗^*P* = .0006; ^∗∗∗∗^*P* < .0001 by 1-way ANOVA with Tukey’s post hoc test.

**Figure 8.**
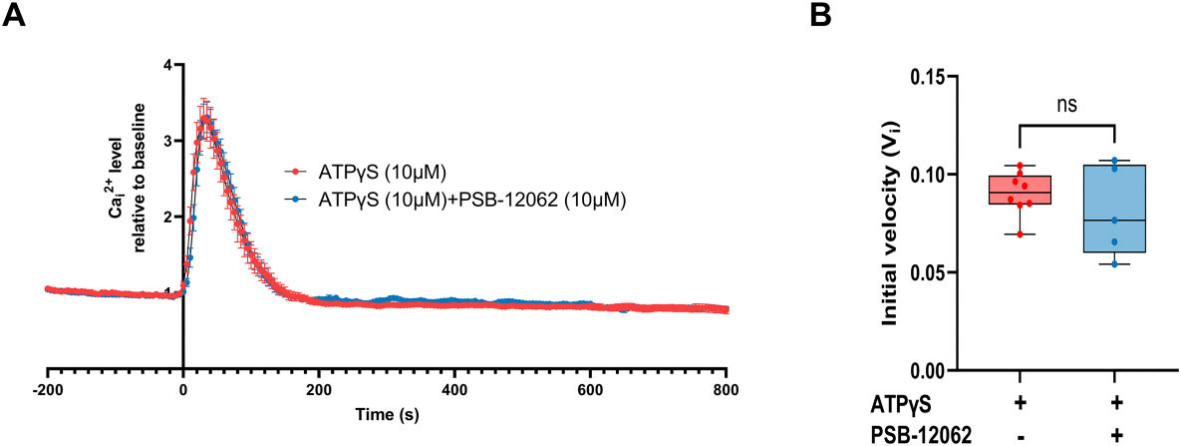
ATP-gated P2X4 is not involved in the ATPγS-mediated Ca^2+^_i_ increase in DC2 cells. (A) Ca^2+^_i_ response after stimulation with 10 µm ATPγS was measured over time in the absence or presence of the selective P2X4 receptor antagonist PSB-12062 (10 µm). (B) No difference was observed when comparing the maximal Ca^2+^_i_ response in the absence or presence of PSB-12062. Data are shown as box and whiskers (min to max); *n* = 5–8.

Stimulation of DC2 cells with extracellular ATPγS (Figure 7A; 10 µm) or BzATP (Figure 7B; 100 µm), a selective P2X7 agonist, both triggered a strong intracellular Ca^2+^_i_ increase. In contrast, pretreatment with PPADS (100 µm), a broad-spectrum P2 receptor antagonist, and A438079 (75 µm), a selective P2X7 receptor antagonist, significantly reduced the Ca^2+^_i_ increase induced by 10 µm ATPγS (Figure 7A). In addition, a similar reduction was observed on the initial velocity (V_i_) of the ATP-evoked Ca^2+^_i_ response in the presence of PPADS and A438079 (Figure 7C and D), confirming the functional role of purinergic P2X7 receptors in ATP-induced calcium signaling in PCs. Furthermore, no effect was observed when comparing the calcium response induced by 10 µm ATPγS in the absence (ATPγS; red trace) or presence of PSB-12062 (10 µm; blue trace), a specific P2X4 antagonist (Figure 8).

### Luminal ATP Regulates NHE3 Trafficking in PCs of the Cauda Epididymis

**Figure 9.**
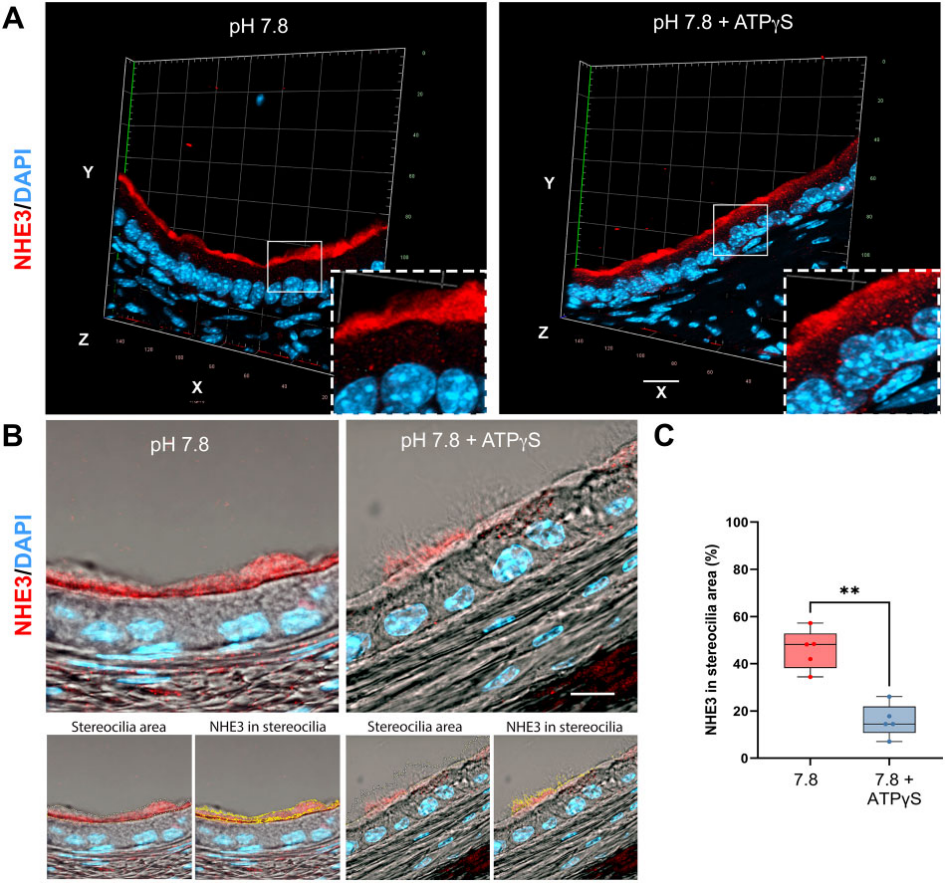
Role of luminal ATP in the regulation of sodium-hydrogen exchanger 3 (NHE3) trafficking in principal cells (PCs) of cauda epididymis. (A) 3D confocal microscopy reconstruction from the cauda epididymis perfused at the alkaline pH of 7.8 in the absence (left panel) or presence of 100 μm ATPγS (right panel). NHE3 is labeled in red and nuclei are visualized in blue with DAPI. Images were acquired with a 20X/0.8 objective. Each box = 20 µm^2^. (B) Single confocal images for NHE3 (red) merged with bright-field images from cauda epididymis perfused at pH 7.8 or pH 7.8 + ATPγS (top panels). The white drawing shows the stereocilia area (bottom panels; stereocilia area). The yellow lines show the selected NHE3-associated pixels within stereocilia (bottom panels; NHE3 in stereocilia). Images were taken with a 40X/1.4 objective with a digital zoom of 2.8. Scale bar = 10 µm. (C) A significant decrease in the area occupied by NHE3 in the stereocilia was observed at pH 7.8 + ATPγS when compared to pH 7.8 alone. The values are expressed as percentage of NHE3-associated area versus the stereocilia area. Data are shown as box and whiskers (min to max); *n* = 5. Each dot represents one epididymis. ***P = .0003 by the Mann-Whitney nonparametric test.

We then investigated the role of ATP-mediated purinergic signaling in modulating NHE3 trafficking in PCs. To do so, the distal cauda epididymis was luminally perfused *in vivo* with ATPγS (100 µm). 3D confocal microscopy reconstruction was first used to evaluate the effect of ATPγS on the subcellular localization of NHE3 at the alkaline pH of 7.8, a condition that induces the accumulation of NHE3 in apical stereocilia.^40,41^ As shown in Figure 9A, when the cauda was perfused at the alkaline luminal pH of 7.8, we confirmed that NHE3 was predominantly located in apical stereocilia, with minimal labeling in the apical intracellular region (left panel). In contrast, when the cauda was perfused with ATPγS at the same alkaline conditions, NHE3 was primarily localized in the apical region of PCs, with little labeling in apical stereocilia (right panel).

Finally, we quantified the effect of ATPγS on NHE3 localization within PC apical stereocilia by using single-Z confocal immunofluorescence images focused on NHE3 in stereocilia and merged with bright-field images to visualize the stereocilia, as we recently published^40^ (Figure 9B). A significant reduction in the area occupied by NHE3 within stereocilia was observed following perfusion with ATPγS at pH 7.8 compared to pH 7.8 alone (Figure 9C), showing that ATPγS promotes NHE3 endocytosis under these conditions.

## Discussion

Previous studies from our group demonstrated that luminal ATP, secreted by PCs, is part of a complex intercellular communication network that ultimately regulates proton secretion by neighboring CCs, thereby contributing to luminal acidification in the epididymis.^22,30,32^ An acidic luminal microenvironment (pH 6.6) in the epididymis is essential for the establishment and maintenance of male fertility as it participates in the acquisition of spermatozoa fertilizing function during their transit in this organ.^28,29,33–39^ However, the molecular machinery employed by epithelial cells to maintain such an acidic luminal environment remained incompletely characterized. This study addresses this gap in knowledge by decoding the molecular players involved in the autocrine regulation of PCs by local ATP signaling. We first characterized the transport mechanisms involved in ATP release by PCs, and then determined the effect of ATP on intracellular calcium signaling and sodium-proton exchange in PCs.

ATP release is highly dependent on mechanical stress and luminal flow.^45,70–72^ In our study, constitutive ATP release was assessed in DC2 cells exposed to minimal mechanical disturbances, by gently adding a small volume of the vehicle or the antagonists into the dish. Additionally, during the intracellular calcium measurements, ATPγS was added to the microfluidic chamber at a minimal and constant flow rate to minimize the influence of shear stress on the response. As we previously described,^30^ we confirm here that DC2 cells constitutively secrete ATP into the extracellular environment. A partial inhibition of ATP release was observed when CFTR, PANX-1, and P2X7 inhibitors were applied separately. While no further inhibition was observed when the inhibitors were applied in pairs, a near-complete inhibition was observed when all 3 inhibitors were applied together. While it is possible that these pathways operate independently and that inhibition of any individual transporter is compensated by the others, these results suggest that CFTR, PANX-1, and P2X7 control ATP secretion, and that all 3 are required to achieve maximal basal ATP secretion. Our data corroborate our previous study demonstrating CFTR involvement in ATP release in the epididymis.^30^ Although CFTR does not act as a direct ATP transporter, it is known to regulate distinct ATP transport pathways.^73^ Our study indicates that such CFTR-regulated ATP pathways are PANX-1 and P2X7 in the epididymis. A limitation of any pharmacological approach is related to the specificity of the inhibitors used. Additional studies using knockdown or knockout approaches would alleviate this concern. However, it remains possible that deleting one transporter might affect the expression of the others. For example, we showed that deletion of CFTR has a strong effect on epithelial differentiation and that it reduces the expression of apical membrane proteins, such as aquaporin 9 and the V-ATPase, in the epididymis.^74^

Among all P2X receptors, P2X7 possesses unique structural and functional properties, including a long cytoplasmic C-terminal tail, and the ability to form a large pore allowing the permeation of large molecules, including nucleotides such as ATP.^75,76^ PANX-1 mediates ATP release through the hexameric assembly of PANX-1 subunits, forming a transmembrane channel (reviewed in Dahl^57^). The interactions between CFTR and PANX-1,^77–79^ as well as between PANX-1 and P2X7, have previously been reported in other tissues.^57,80,81^ Interestingly, it was shown that ATP released into the extracellular space by PANX-1 could activate P2X7, which in turn could activate PANX-1, providing a positive feedback loop.^57,76,81–83^ This mechanism would ensure a rapid and efficient ATP release in response to cellular demands. Under physiological conditions, this positive feedback mechanism would be counterbalanced by ATP-dependent inhibition of PANX-1. Indeed, high ATP concentrations induce PANX-1 internalization,^80,84^ while P2X7 is not desensitized at elevated ATP levels and is further activated by prolonged ATP exposure, a process known as facilitation.^85^ Thus, P2X7 was shown not only to participate in physiological processes but also to be a key player under pathophysiological conditions. As such, the presence of P2X7-positive cells in the epididymal interstitium that we report here would support its known role in immune responses.^56,86–89^ While these cells were not identified in this study, it will be interesting to determine whether they are part of the complex network of mononuclear phagocytes previously identified in the epididymis.^90^

Given the involvement of CFTR in PANX-1- and P2X7-mediated ATP release shown in this study, it would be interesting to determine whether CFTR also plays a role in the regulatory feedback mechanisms governing ATP release in the epididymis, and whether CFTR mutations affect these complex interactions. Indeed, CFTR mutations are a leading cause of male infertility and defective ATP signaling in the epididymis might contribute to post-testicular tract dysfunction. Of note, CFTR can be activated by extracellular ATP^91,92^ and, similarly to ATP secretion, CFTR is activated by mechanical stimuli such as cell stretch or shear stress.^93^ Therefore, it is possible that luminal ATP would provide a positive feedback mechanism by stimulating its own release through CFTR activation. Several epithelial tissues, including the epididymis, the kidney, and the intestine, experience variations in luminal flow, which are subjected to mechanical disturbances via either expansive pressure or shear forces.^72,94–96^ Being located in the apical membrane of PCs, CFTR, PANX-1, and P2X7 are suitably positioned to respond to these mechanical stimuli by activating ATP release. It would be interesting to determine in future studies whether shear stress through luminal flow variations could modulate ATP release in DC2 cells or PCs in the intact epididymal epithelium. Altogether, this study highlights the complexity and efficiency of ATP release mechanisms, ensuring that ATP levels remain tightly regulated to support cellular functions.

We then examined whether PCs could be modulated in an autocrine manner by luminal ATP. The local extracellular ATP concentration near epithelial cells is highly dependent on the activity of ectonucleotidases that are anchored on their apical membrane.^32,45,97,98^ In our study, we used ATPγS, the nonhydrolyzable form of ATP, to specifically examine its direct role on the response of PCs. Addition of ATPγS into the culture medium induced a dose-dependent increase in Ca^2+^_i_ in DC2 cells. While a maximal response was observed at 100 µm, Hill equation analysis showed that ATP triggers a Ca^2+^_i_ increase with an EC_50_ of 10 µm. The activity of ectonucleotidases depends on luminal pH in the epididymis, which would provide a feedback mechanism linking pH and local ATP signaling pathway to control proton secretion by epithelial cells.^22^ The maximal increase in Ca^2+^_i_ that we observed at 100 µm ATPγS was significantly reduced in the absence of extracellular calcium, indicating the participation of P2X receptors. Inhibition of P2X7 at 10 µm ATPγS significantly reduced the Ca^2+^_i_ increase, whereas P2X4 inhibition had no effect. Furthermore, P2X7-specific activation by BzATP induced a robust Ca^2+^_i_ increase, further supporting its role in this ATP-dependent calcium response. The remaining calcium increase observed in the absence of extracellular calcium suggests the participation of P2Y receptors, which induce the release of Ca^2+^ from intracellular stores. While the specific P2Y receptors were not identified in this study, P2Y2 and P2Y4, which are expressed in DC2 cells and in the epididymis, and whose preferred agonist is ATP,^2^ are likely candidates. Typically, P2 receptors require approximately 1–10 µm of ATP to reach full activation.^45^ Among these receptors, P2X7 was initially thought to be inactive at low ATP concentrations, becoming active only under pathophysiological conditions where high concentrations of ATP are reached. However, our findings suggest that P2X7 participates in Ca^2+^_i_ increase at 10 µM ATPγS, indicating that it could regulate epithelial cell functions under physiological conditions.

To further characterize the role of ATP signaling in PCs, we performed functional analysis on the intact epididymis. We previously showed that NHE3, located on the apical membrane of PCs, contributes to luminal acidification.^41^ NHE3 is regulated by recycling mechanisms, and its localization at the cell surface depends on several factors, including intracellular calcium. In particular, PKC-dependent phosphorylation has been shown to induce NHE3 internalization in other tissues, such as the kidney and intestine.^42^ Given the ATPγS-induced Ca^2+^_i_ increase that we observed in PCs, we investigated whether local ATP signaling regulates NHE3 subcellular localization. To better reveal the potential ATP-induced NHE3 internalization, we perfused the lumen of the epididymis at an alkaline pH of 7.8, a condition that promotes NHE3 accumulation at the apical membrane.^40,41^ Under these conditions, luminal ATPγS significantly reduced NHE3 localization within apical stereocilia, favoring its redistribution into the intracellular apical region. This result shows that ATP signaling overrides the alkaline pH–dependent accumulation of NHE3 in stereocilia.^40,41^ Further studies will be required to identify the purinergic receptor involved in this response, and to determine whether PKC-dependent phosphorylation mediates NHE3 internalization. These observations expand on our previous work, demonstrating that adenosine, a product of ATP hydrolysis, regulates NHE3 cell surface expression in epididymal PCs. Specifically, ADORA2B receptor promotes NHE3 internalization, while ADORA3 maintains NHE3 in apical stereocilia.^40^ Together, our findings highlight an autocrine regulatory mechanism through which local ATP secretion by PCs, via local purinergic signaling, modulates NHE3 cell surface expression, either directly by ATP itself, or through its hydrolysis product adenosine.

**Figure 10.**
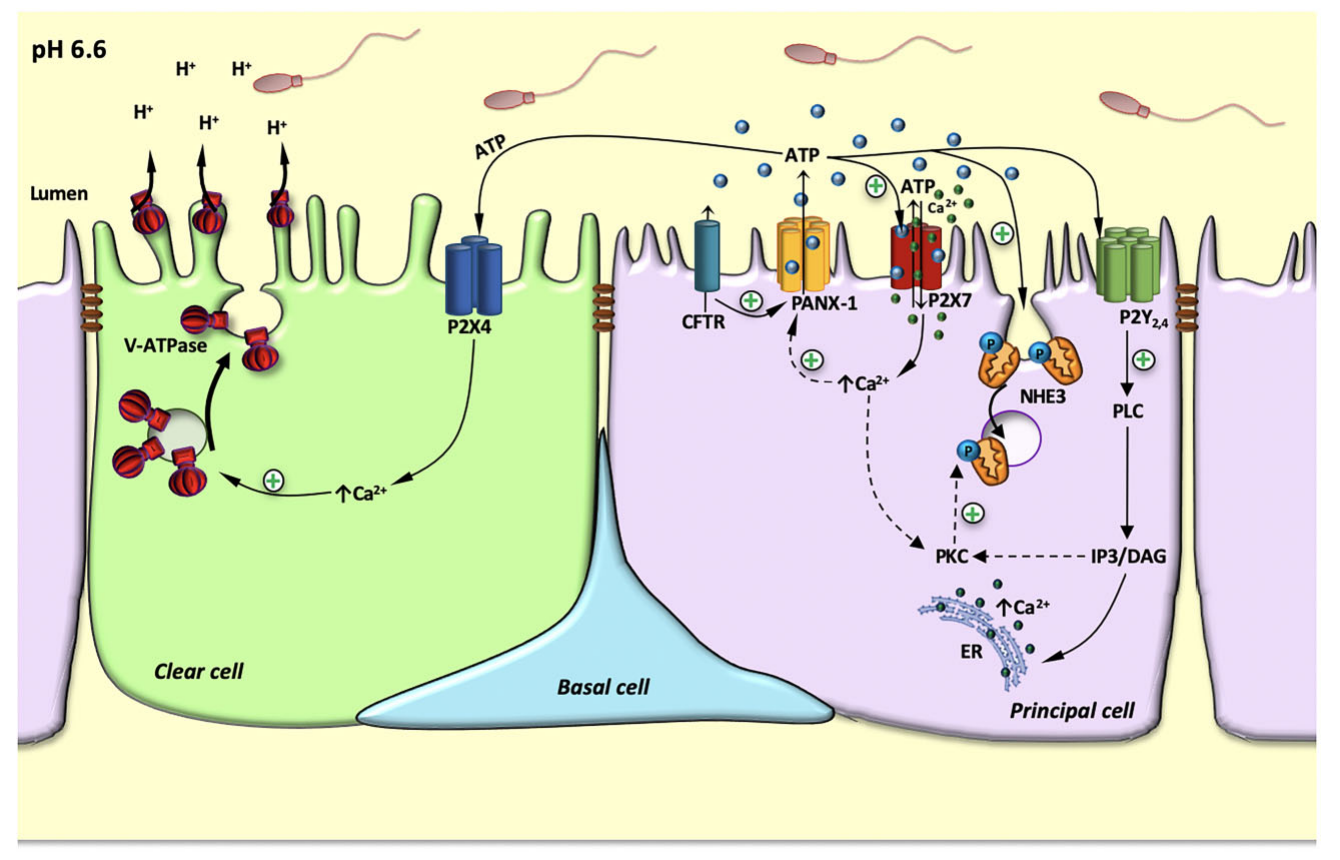
Proposed model for the ATP-dependent paracrine and autocrine regulation of proton secretion by clear cells (CCs) and principal cells (PCs), respectively. PCs constitutively secrete ATP into the lumen through the concerted interaction between cystic fibrosis transmembrane conductance regulator (CFTR), PANX-1, and pannexin 1 (P2X7). Activation of P2X7 by luminal ATP further activates PANX-1, providing a positive feedback mechanism for ATP release. P2X7 is an ATP-gated receptor that induces the entry of Ca^2+^ from the luminal space into the cell. P2Y receptors were also identified in PCs. Upon activation with luminal ATP, these GPCR receptors induce the release of calcium from intracellular stores. *In vivo* luminal perfusion of the cauda epididymis with ATPγS, the nonhydrolyzable form of ATP, induced the internalization of NHE3 from the apical membrane of PCs, showing the autocrine regulation of PCs. In a previous study,^22^ we showed that PANX-1-dependent ATP secretion by PCs leads to the activation of P2X4 located on the apical membrane of CCs, followed by the apical membrane accumulation of the proton pump V-ATPase. Thus, a complex ATP local signaling pathway exists in the epididymis, for the autocrine and paracrine regulation of luminal acidification in the epididymis. The full arrows indicate the results obtained in the present or previous^22,32^ studies. The dashed arrows indicate proposed mechanisms of action.

Local luminal ATP signaling is part of a global purinergic network that either acts in a paracrine manner to regulate V-ATPase-dependent proton secretion by CCs,^22,32^ or participates in the autocrine regulation of NHE3-dependent sodium/proton exchange by PCs (this study and Belardin^40^) (Figure 10). Our previous study demonstrated that luminal ATPγS induces V-ATPase apical accumulation in CCs via P2X4 activation, and PANX-1 participates in the alkaline pH–dependent V-ATPase accumulation in CCs.^22^ These results highlight the complexity of purinergic signaling for the control of luminal pH in the epididymis. While P2X4 activation by luminal ATP stimulated proton secretion by CCs,^22^ our current study reveals that P2X7 activation in PCs induces the opposite effect by promoting NHE3 internalization. The contrasting effects of ATP on different epithelial cell types that are adjacent to each other remains unexplained. The answer might rely on ATP hydrolysis by ectonucleotidases to form adenosine, a process that is activated at alkaline luminal pH. Thus, additional studies will be required to fully dissect the respective roles of ATP and adenosine in the regulation of proton secretion by CCs and PCs. On the other hand, P2X7 was recently shown to be modulated by extracellular pH,^99^ bringing another level of complexity to the control of luminal acidification by purinergic signaling.

Of note, P2X7 is not only involved in ATP secretion and intracellular calcium signaling, but its activation also mediates sodium influx and potassium efflux.^100,101^ Given that NHE3 participates in sodium influx in exchange for protons, future studies should investigate the roles of P2X7 and NHE3 in the establishment of the unique epididymal luminal environment, which is characterized by high potassium and low sodium concentrations, contributing to sperm quiescence during their storage period. For example, it is possible that the internalization of NHE3 induced by ATP, a condition that activates proton secretion by CCs, might protect the epididymis from an acid load–induced Na^+^ secretion.

In summary, we show here that luminal ATP signaling participates in the autocrine regulation of PCs through the combined action of CFTR, PANX-1, and P2X7 for the release of ATP, which then activates intracellular calcium signaling and promotes NHE3 internalization. This study enhances our understanding of purinergic signaling in the epididymis and may provide insights into idiopathic causes of male infertility related to post-testicular sperm maturation. Since local purinergic signaling is involved in the regulation of transepithelial transport in several tubular organs, including the kidney and intestine, our findings provide novel insights into how epithelial cells sense and respond to local luminal ATP to modulate their respective functions. Given that purinergic signaling is an emerging therapeutic target for several diseases,^12,102–104^ our study also highlights the potential consequences of these new therapeutic interventions on male reproductive health.

## Data Availability

All data pertaining to this article are available within the main document.
